# The IL-21/IL-21R signaling axis regulates CD4^+^ T-cell responsiveness to IL-12 to promote bacterial-induced colitis

**DOI:** 10.1093/jleuko/qiae069

**Published:** 2024-03-18

**Authors:** Shahram Solaymani-Mohammadi

**Affiliations:** Laboratory of Mucosal Immunology, Department of Biomedical Sciences, School of Medicine and Health Sciences, University of North Dakota, 1301 North Columbia Road, Suite W315, Stop 9037, Grand Forks, ND, United States

**Keywords:** CD4+ T cells, *Citrobacter rodentium*, colitis, IL-12Rβ2, IL-21, inflammation, intestine

## Abstract

IL-21/IL-21R signaling dysregulation is linked to multiple chronic intestinal inflammatory disorders in humans and animal models of human diseases. In addition to its critical requirement for the generation and development of germinal center B cells, IL-21/IL-21R signaling can also regulate the effector functions of a variety of T-cell subsets. The antibody-mediated abrogation of IL-21/IL-21R signaling led to the impaired expression of IFN-γ by mucosal CD4^+^ T cells from human subjects with colitis, suggesting an IL-21/IL-21R–triggered positive feedback loop of the T_H1_ immune response in the colon. Despite recent advances in our understanding of the mechanisms underpinning the regulation of proinflammatory immune responses by the IL-21/IL-21R signaling axis, it remains unclear how this pathway or its downstream molecules contribute to inflammation during bacterial-induced colitis. This study found that IL-21 enhances the surface expression of IL-12Rβ2, but not IL-12Rβ1, in CD4^+^ T cells, leading to T_H1_ differentiation and stability. Consistently, these findings also point to an indispensable role of the IL-12Rβ2 signaling axis in promoting proinflammatory immune responses during *Citrobacter rodentium*–induced colitis. Genetic deletion of the IL-12Rβ2 signaling pathway led to the attenuation of *C. rodentium*–induced colitis in vivo. The genetic deletion of the IL-12Rβ2 signaling pathway did not alter the host's ability to respond adequately to *C. rodentium* infection or the ability of *Il12rb2*^−/−^ mice to express antigen-specific cytokines (IFN-γ, IL-17A). IL-21 is a pleiotropic cytokine exerting a wide range of immunomodulatory functions in multiple tissues, and its direct targeting may result in undesirable off-target consequences. These findings highlight the possibility for targeted manipulations of signaling cascades downstream of main regulators of proinflammatory responses to control invading pathogens while preserving the integrity of host immune responses. A better understanding of the novel mechanisms by which IL-21/IL-21R signaling regulates bacterial-induced colitis will provide insights into the development of new therapeutic and preventive strategies to harness IL-21/IL-21R signaling or its downstream molecules to treat infectious colitis.

## Teaser

IL-21/IL-21R regulation of colitis.

## 1. Introduction

As a member of the common gamma-chain (γc) signaling family of cytokines, IL-21 plays critical roles in the regulation of various biological functions such as proliferation, development, and function/differentiation of both the innate and adaptive arms of the immune system.^1–3^ IL-21 is a pleiotropic cytokine expressed by CD4^+^ T cells, primarily follicular helper (T_FH_) cells, and it mainly activates STAT3 and, to a lesser extent, STAT1 and STAT5.^4–8^ The phosphorylated STAT molecules dimerize and translocate into the nucleus, inducing the expression of a set of downstream target genes, commonly known as the IL-21 target genes.^1,2,6–8^ While IL-21 is almost exclusively expressed by mucosal colonic lamina propria (cLP) CD4^+^ T cells during colitis,^6,9^ the IL-21 cognate receptor, IL-21R, is expressed by multiple cell subsets of hematopoietic origin, including CD4^+^ T cells, dendritic cells (DCs), natural killer cells, macrophages, and neutrophils.^6,10,11^

Several lines of evidence suggest that IL-21 signaling modulates the effector functions (e.g. cytokine production) of several T-cell subsets.^6,12–16^ Individuals with colitis and animal models of colonic inflammation show dysregulated expression of IL-21.^17–21^ IL-21 can exacerbate colonic inflammation by augmenting STAT4 expression and enhancing IFN-γ release by T cells.^12^ Consistent with these findings, the IL-21/IL-21R axis blockade impaired the release of IFN-γ by mucosal lymphocytes, resulting in attenuated colonic inflammation.^12^ These observations point to a positive feedback loop mediated by the IL-21/IL-21R axis that could amplify an inflammatory T_H1_ response at the mucosal surfaces of the colon.^12^ To this end, mice lacking a functional IL-21/IL-21R signaling axis (*Il21r*^−/−^) displayed significantly reduced colitis as compared with their wild-type (WT) controls. This suggests that the antibody-based blockade of the IL-21/IL-21R axis or its downstream signaling molecules could be devised as novel alternative strategies to prevent intestinal inflammation.

IL-12 is one of the most critical cytokines required for the development and polarization of a T_H1_-biased proinflammatory immune response profile.^22–26^ The cytokine IL-12 consists of 2 subunits, IL-12p35 and IL-12p40, linked by disulfide bonds to form a heterodimeric cytokine.^24,27–29^ This cytokine exerts its potent proinflammatory functions through binding to its 2 cognate receptors, IL-12Rβ1 and IL-12Rβ2.^30,31^ IL-12Rβ1 is constitutively expressed in naive T cells, while IL-12Rβ2 expression is inducible and necessary for T-cell activation and polarization toward a T_H1_-inducing milieu.^24,32,33^ IL-12Rβ2's interaction with STAT4 enhances its expression and further stabilizes T_H1_-polarized immune responses by increasing T-cell responsiveness to IL-12.^32,33^ Furthermore, IL-12 can promote the phosphorylation of STAT4 in T cells, resulting in the regulation of T-cell effector functions, including the release of multiple proinflammatory cytokines.^32,33^ IL-12 was found to be overexpressed in mucosal samples of human subjects with inflammatory bowel disease (IBD), which is consistent with its inflammation-promoting functions.^34–38^ Likewise, the expression of IL-12Rβ2, but not IL-12Rβ1, was significantly upregulated in human subjects with active Crohn disease (CD), but not in those with inactive disease or healthy controls.^38^

Multiple cytokines can efficiently drive a proinflammatory immune profile by increasing the responsiveness to IL-12, thereby promoting T_H1_ differentiation and stabilization in T cells.^39–42^ It is currently unclear how a functional IL-21/IL-21R regulates colonic inflammation by promoting CD4^+^ T-cell responsiveness to IL-12. While the cascade of events leading to the activation of STAT3 by IL-21 has been extensively studied,^43^ it is unclear how the events downstream of IL-12Rβ2 signaling affect the expression of proinflammatory gene profiles during colitis. Using a well-established and physiologically relevant murine model of *Citrobacter rodentium*–induced colitis, the mechanisms by which the IL-21/IL-21R signaling axis regulates colitis by enhancing the IL-12 responsiveness of CD4^+^ T cells were explored. Consistent with our prior observations pointing to the requirement of intact IL-21/IL-21R signaling in the regulation of colitis following *C. rodentium* infection,^6^ the expression of IFN-γ and IL-12Rβ2, but not IL-12Rβ1, was impaired in distal cLP CD4^+^ T cells of *Il21r*^−/−^ mice following *C. rodentium* infection. The impaired expression levels of *Il12rb2* and *Ifng* transcripts were associated with significantly lower inflammatory scores in the distal colon of *Il21r*^−/−^ mice as compared with WT controls. Mice lacking functional IL-12Rβ2 signaling exhibited attenuated colitis following *C. rodentium* infection, whereas the ability of those mice to effectively eradicate *C. rodentium* infection was unaffected. Overall, these findings demonstrate that the IL-12/IL-12R signaling pathway plays a central role in mediating the pathogenesis of colitis by regulating the effector functions of mucosal CD4^+^ T cells.

## 2. Materials and methods

### 2.1 Ethics statement

All animal experiments were performed at the Association for Assessment and Accreditation of Laboratory Animal Care (AAALAC)-accredited Center for Biomedical Research at the University of North Dakota according to procedures approved by the Institutional Biosafety Committee (IBC-202008-004), following guidelines and protocols approved by the University of North Dakota Animal Care and Use Committee (2007-2, 2008-6, 2308-0049, 2310-0059) in compliance with the National Institutes of Health guidelines.

### 2.2 Mice

Age- and sex-matched 6- to 8-wk-old female and male littermates were used in all experiments. C57BL/6J and B6.129S1-*Il12rb2*^tm1Jm^/J (#003248) and B6.129-*Il21r^tm1Kopf^*^/^J (#019115)^6^ on a C57BL/6J background were purchased from the Jackson Laboratory. Mice were genotyped using genomic DNA isolated from tail snips.

### 2.3 Cell cultures and cytokines

Splenic CD4^+^ T cells were negatively purified from age- and sex-matched naive (uninfected) *Il21r*^−/*−*^ mice and their WT controls using the MojoSort™ Mouse CD4 T Cell Isolation Kit (BioLegend) following the manufacturer's instructions. The purity of isolated CD4^+^ T cells was ≥97%. A total of 2 × 10^6^ purified splenic CD4^+^ T cells were seeded in duplicate in 1 mL complete RPMI 1640 medium (Gibco), supplemented with 10% fetal bovine serum (FBS; R&D Systems) and 100 μg/mL penicillin/streptomycin (Gibco). Purified CD4^+^ T cells were allowed to rest in a 5% CO_2_ incubator for 2 h at 37 °C before being treated with 20 ng/mL rmIL-21 (Peprotech). Untreated (i.e. no rmIL-21 treatment) purified CD4^+^ T cells served as negative controls. The stimulated cells were harvested after 1 h and 4 h by centrifugation at 900 × *g* for 5 min and washed twice with ice-cold 1× phosphate-buffered saline (PBS), followed by the total RNA extraction using the RNeasy Micro Kit (Qiagen) for Nanostring analysis.

### 2.4 Infection and bacterial burden quantification

*C*. *rodentium* strain DBS100 (ATCC 51459) was obtained from the American Type Culture Collection and cultured in Luria–Bertani (LB; Sigma-Aldrich) broth at 37 °C overnight with shaking at 200 rpm. Small aliquots (200 µL) of the overnight bacterial cultures were transferred into 50 mL Falcon® tubes (BD Falcon) containing fresh LB medium and were further propagated at 37 °C with shaking at 200 rpm. Bacterial cultures were grown to an optical density of 0.8 at 600 nm (OD600; Spectramax Plus 384 spectrophotometer; Molecular Devices), then pelleted by centrifugation at 4,500 rpm for 20 min at 4 °C. The pelleted bacteria were resuspended in 1× PBS (pH 7.4) and further adjusted at a concentration of 5 × 10^9^ colony-forming units (CFU)/mL. Mice were orally gavaged with 100 µL bacterial stock, containing 5 × 10^8^ CFU of *C. rodentium* per mouse.^6,44,45^

An ovalbumin (OVA)–expressing *C. rodentium* (OVA-*Citrobacter*) strain possessing a kanamycin resistance gene (provided by Dr. Daniel Mucida at Rockefeller University) was utilized in some experiments to measure antigen-specific immune responses in cLP CD4^+^ T cells of infected animals.^6,46^ Mice were administrated kanamycin sulfate (Gibco) in drinking water (1 g/L) ad libitum, starting 4 d before bacterial inoculation and throughout the entire duration of the experiments, to prevent loss of OVA expression. The drinking water containing fresh kanamycin was replenished every other day.

Mice were individually placed in clean cages with no bedding at different time points after infection. Fecal pellets (100 to 200 mg) were collected, weighed, homogenized in 2 mL sterile PBS, serially diluted 10-fold, and plated onto MacConkey agar (BD Difco) without or with kanamycin (50 to 100 µg/mL). Inoculated agar plates were incubated at 37 °C for 24 h, and the bacterial numbers were counted and expressed as CFU/g feces.

Bacterial translocation into extraintestinal sites, including spleens and livers, was assessed by plating serially diluted tissue homogenates onto MacConkey agar, as previously described.^6,44^ The bacterial burdens in extraintestinal tissues were expressed as CFU/g tissue.

### 2.5 Histological examinations

*C. rodentium*–infected colons were cleaned of fecal contents, rolled up into a Swiss roll configuration (proximal to distal), fixed in 10% buffered formalin for 24 h, transferred to 70% ethanol, and processed into paraffin blocks as previously described.^6,44^ Colon tissue sections (5 µm thick) were stained with hematoxylin and eosin, and the severity of colitis was evaluated by a blinded observer using a scoring system described earlier.^47^ This scoring system uses an 0 to 4 scale to evaluate parameters such as crypt architecture loss, ulceration, inflammatory cell infiltration, muscle thickening, goblet cell depletion, and the formation of crypt abscesses, with 0 being normal, 1 being minimal, 2 being mild, 3 being moderate, and 4 being marked.

### 2.6 Isolation of the colon lamina propria lymphocytes

Colon lamina propria lymphocytes (LPLs) were isolated as described earlier.^6,44^ Briefly, colons of naive (uninfected), and infected mice were aseptically removed, opened longitudinally, cleaned of feces, and rinsed with ice-cold 1× PBS. The colon tissues were further minced into smaller pieces (0.5 cm), digested in serum-free RPMI 1640 medium containing 50 μg/mL Liberase™ TL Research Grade (Roche), and 0.1 mg/mL DNase (Roche) and supplemented with penicillin/streptomycin for 45 min at 37 °C.^6,44^ The LPLs were released from digested tissues with continuous vortexing and passed through 70-μm cell strainers (BD Falcon). The isolated LPLs were washed twice with ice-cold 1× PBS and counted using a hemocytometer. The LPLs were then fluorescence-activated cell sorting (FACS)-sorted into hematopoietic (CD4^+^ T cells, neutrophils, macrophages, and DCs) and nonhematopoietic (colonic intestinal epithelial cells [cIECs]) subtypes using a BD FACSAria cell sorter (BD) as previously described.^6^

### 2.7 Flow cytometry and intercellular cytokine staining

The Fc receptor blockade was performed by incubating colon LPL single-cell suspensions with 1 µg/10^6^ cells of TruStain FcX™ anti-mouse CD16/CD32 (clone 93; BioLegend) in FACS buffer (3% FBS in ice-cold 1× PBS) at 4 °C for 20 min. LPLs then were stained with the fixable Ghost Dye™ Red 780 viability dye (Tonbo Biosciences) in protein/serum-free ice-cold 1× PBS for 20 min at 4 °C in the dark following the manufacturer's instructions. The surface staining was performed in duplicate in FACS buffer using a cocktail of mouse monoclonal antibodies, including anti-CD3-BV421 (clone 17A2), anti–CD4-Alexa Fluor 488 (clone GK1.5), anti–CD326-PE-Cy7 (clone G8.8), anti–F4/80-Brilliant Violet 421 (clone BM8), anti–CD11b-APC (clone M1/70), anti–major histocompatibility complex (MHC) class II (I-A/I-E)–Pacific Blue (clone M5/114.15.2), and anti–CD45-PE/Cy7 (clone 30-F11) (all from BioLegend) and anti–CD11c-Texas Red (clone MCD11C17; Invitrogen). The surface expression of IL-21R in cLP CD4^+^ T cells was assessed by using a biotinylated anti–IL-21R antibody (eBio4A9; eBioscience), followed by staining with PE-streptavidin (BioLegend).

For measuring the intracellular expression of cytokines by antigen-specific CD4^+^ T cells in response to OVA, the colonic LPLs isolated from OVA-*Citrobacter*–infected mice 9 d after infection were restimulated ex vivo for 8 h with purified chicken egg white OVA (100 μg/mL; Sigma-Aldrich), adding brefeldin A at 10 μg/mL (BioLegend) for the last 6 h. The restimulated cells were then surface stained, fixed, and permeabilized using the Foxp3 Transcription Factor Staining Buffer Set (eBioscience). They were stained with anti-mouse IFN-γ-PE (clone XMG1.2; BioLegend) and anti-mouse IL-17A–APC (clone T11-18H10.1; BioLegend) monoclonal antibodies as described.^6^ Data were acquired using a BD FACS Symphony A3 flow cytometer (BD Biosciences) and were analyzed using FlowJo software (v10.0.7.2; Tree Star).

### 2.8 Ex vivo colon culture and enzyme-linked immunosorbent assay

The colons of infected mice were aseptically removed, opened longitudinally, cleaned of fat and connective tissues, and sliced into smaller pieces, as described.^6^ Colonic tissues were rinsed twice in ice-cold PBS and cultured in 48 well-plates in complete RPMI 1640 medium (Gibco), supplemented with 10% FBS (R&D Systems) and 100 μg/mL penicillin/streptomycin (Gibco) at 37 °C in a 5% CO_2_ incubator for 24 h. The colon culture supernatants were collected, cleared by centrifugation at 14,000 rpm for 5 min, and used for cytokine analysis using a mouse IL-12p35 enzyme-linked immunosorbent assay kit (Abbexa). The optical density was measured at 450 nm using a Synergy HT microplate reader (BioTek). Results were expressed as picograms per gram (pg/g) of colon.

### 2.9 Nanostring analysis

The colons were aseptically removed, opened longitudinally, and cleaned of fat, connective tissues, and fecal materials. Total RNA was isolated from the whole distal colon tissues of age- and sex-matched naive (uninfected) and infected (day 9 post infection [p.i.]) *Il21r*^−/*−*^ mice and their WT controls,^6^ as well as *Il12rb2*^−/*−*^ mice and their *Il12rb2*^+/+^ littermate controls, using a Qiagen RNeasy Plus Mini Kit and QIAshredder spin columns (Qiagen) per the manufacturer's instructions. The total RNA concentration and purity were assessed, and a total of 100 ng purified RNA extracted from colonic tissues was subjected to gene expression analysis using the nCounter® Mouse Host Response Panel (Nanostring Technologies). This panel includes 785 genes across 50 pathways, along with 12 internal control genes to normalize the data. The Nanostring nSolver™ analysis software (v4.0) was employed to analyze the resource compiler (RCC) files.

### 2.10 Gene ontology and pathway analysis

The gene ontology classifications and functional enrichment of the host response gene signature were performed using a gene panel algorithm provided by the Nanostring platform. The normalized Nanostring values were used to construct the heatmaps using the web-based open tool Morpheus (http://software.broadinstitute.org/morpheus).

### 2.11 Statistical analysis

The analysis of the data was performed using GraphPad Prism (v9.5.0 for Windows; GraphPad Software) and presented as the mean ± SEM. A 2-tailed unpaired Mann–Whitney *U* test or a 1-way analysis of variance, followed by a Bonferroni post hoc adjustment test for multiple comparisons, was utilized. Statistical significance was defined as *P < 0.05.

## 3. Results

### 3.1 The expression of *Ifng* and *Il12rb2*, but not *Il12rb1*, is impaired in colonic CD4^+^ T cells of *Il21r*^−/−^ mice during colitis

**Fig. 1.**
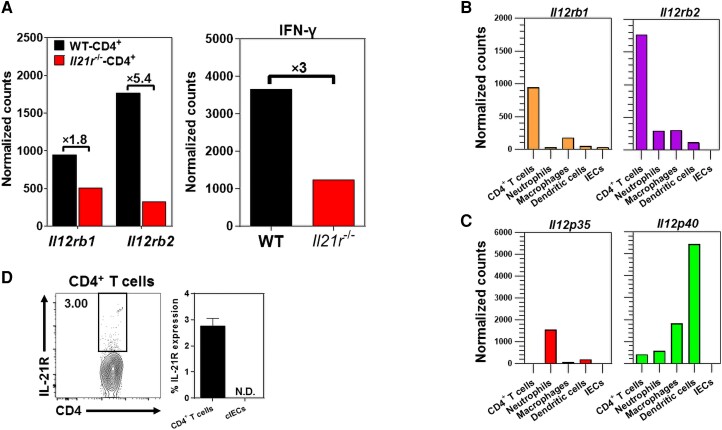
A functional IL-21/IL-21R signaling is required for the optimal expression of *Il12rb2* and *Ifng* in colonic CD4^+^ T cells. (A) The expression of *Il12rb1*, *Il12rb2*, and *Ifng* by distal cLP CD4^+^ T cells, defined as CD45^+^CD3^+^CD4^+^ cells, in *Il21r^−^*^/*−*^ mice following *C. rodentium* infection. (B, C) The expression of *Il12rb1/Il12p40* and *Il12rb2/Il12p35* by FACS-sorted distal cLPLs of age- and sex-matched female C57BL6 mice 9 d after *C. rodentium* infection, as determined by Nanostring analysis. The hematopoietic cell types included CD4^+^ T cells (CD326^−^CD45^+^CD3^+^CD4^+^), neutrophils (CD326^−^CD45^+^CD11b^+^Ly6G^+^), macrophages (CD326^−^CD45^+^CD11b^+^F4/80^+^), and dendritic cells (CD326^−^CD45^+^CD11c^+^MHC-II^+^), whereas cells of the nonhematopoietic origin (cIECs) were defined as CD326^+^CD45^−^ subtypes. The results (A to C) are pooled data from 25 to 30 mice. (D) Flowcytometric analysis of IL-21R surface expression in distal cLP CD4^+^ T cells and cIECs in naive (uninfected) C57BL/6 mice (*n* = 5). N.D., not detected.

Mice lacking functional IL-21/IL-21R signaling (*Il21r*^−/−^) showed attenuated *C. rodentium*–induced colitis, despite significantly greater fecal bacterial loads than their WT counterparts.^6^ It was hypothesized that attenuated colitis in *Il21r*^−/−^ mice could be due to their inability to optimally mount a proinflammatory T_H1_-biased immunity in response to *C. rodentium* infection in the colon. In an unbiased screen for genes that were impaired in cLP CD4^+^ T cells of *Il21r*^−/−^ mice during *C. rodentium* infection,^6^ the expression of *Il12rb1* and *Il12rb2* was shown to be impaired in FACS-sorted cLP CD4^+^ T cells isolated from *Il21r*^−/−^ mice as compared with their WT controls (Fig. 1A). However, impaired expression of *Il12rb2* in cLP CD4^+^ T cells was more pronounced than *Il12rb1* (Fig. 1A). The Nanostring analysis revealed that impaired expression of *Il12rb2* in cLP CD4^+^ T cells of *Il21r*^−/−^ mice correlated with reduced *Ifng* expression in those cells 9 d after *C. rodentium* infection (Fig. 1A). Collectively, these findings indicated a novel and previously unknown role played by IL-21/IL-21R signaling in driving CD4^+^ T-cell responsiveness to IL-12 by enhancing the expression of IL-12Rβ2 in CD4^+^ T cells, thereby imprinting a T_H1_-biased immunity during bacterial-induced colitis.

### 3.2 IL-12Rβ1 and IL-12Rβ2 are predominately expressed by cLP CD4^+^ T cells following *C. rodentium* infection

The expression of IL-12Rβ1 and IL-12Rβ2 by main hematopoietic (CD45^+^EpCAM*^−^*) and nonhematopoietic (CD45^−^EpCAM^+^) subtypes in the cLP was assessed 9 d after *C. rodentium* infection. The Nanostring analysis of the enzymatically isolated FACS-sorted subsets demonstrated that cLP CD4^+^ T cells expressed the highest levels of transcripts for both IL-12Rβ1 and IL-12Rβ2 following *C. rodentium* infection in the distal colon (Fig. 1B). Notably, cLP CD4^+^ T cells expressed more transcripts for IL-12Rβ2 than IL-12Rβ1. Following *C. rodentium* infection, neutrophils and DCs expressed the highest levels of transcripts for IL-12p35 (binding IL-12Rβ2) and IL-12p40 (binding IL-12Rβ1) in the colon LPLs of C57BL/6 mice. The cIECs did not express detectable levels of transcripts for IL-12 (IL-12Rβ1, IL-12Rβ2) or its receptors (IL-12Rβ1, IL-12Rβ2) (Fig. 1C). Altogether, these findings indicated that mucosal CD4^+^ T cells were the major expressors of both IL-12Rβ1 and IL-12Rβ2 transcripts in the cLP following *C. rodentium* infection. Additionally, CD4^+^ T cells were likely the primary targets for the immunomodulatory potency of IL-12 signaling during colitis. In naive (uninfected) mice, 2% to 3% of distal cLP CD4^+^ T cells expressed IL-21R, whereas no surface expression of IL-21R was detected in cIECs under steady-state conditions in uninfected mice (Fig. 1D).

### 3.3 IL-21 selectively induces the surface expression of IL-12Rβ2, but not IL-12Rβ1, in CD4^+^ T cells

**Fig. 2.**
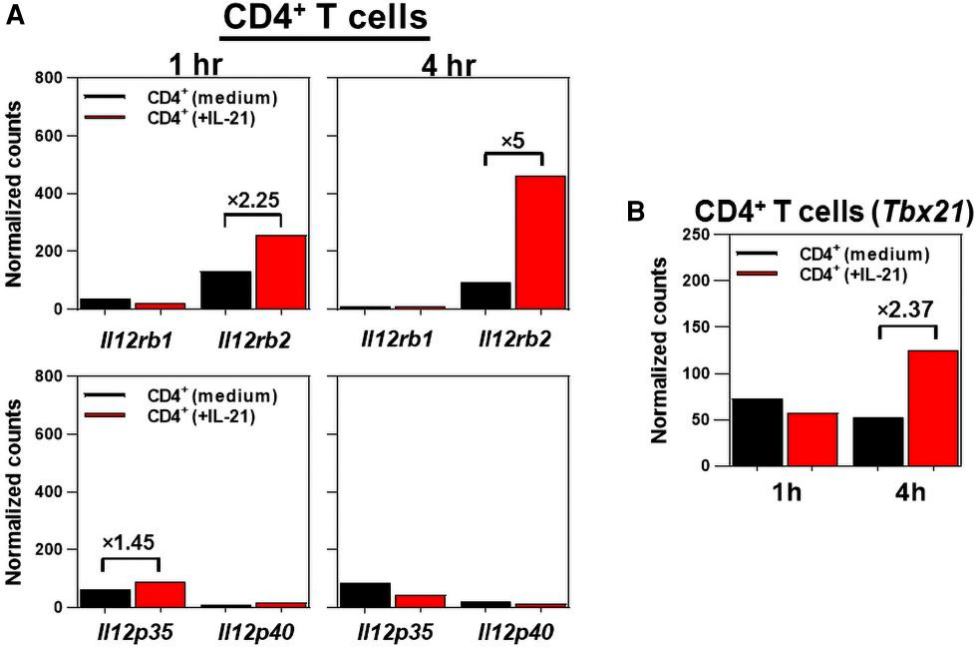
IL-21 selectively induces expression of *Il12rb2*, but not *Il12rb1*, and *Tbx21* (T-bet) in CD4^+^ T cells. (A) Nanostring analysis of *Il12rb1/Il12p40*, *Il2rb2/Il12p35*, and (B) *Tbx21* gene expression after treating negatively selected CD4^+^ T cells (2 × 10^6^/well) with 20 ng/mL recombinant murine IL-21 (rmIL-21) for 1 and 4 h. Control CD4^+^ T cells (2 × 10^6^/well) received medium (no rmIL-21). Results are pooled of CD4^+^ T cells from age- and sex-matched naive (uninfected) C57BL/6 mice (*n* = 5).

The cLP CD4^+^ T cells of *Il21r*^−/−^ mice showed an impaired expression of IL-12Rβ2, but not IL-12Rβ1, 9 d following *C. rodentium* infection (Fig. 1A). It was hypothesized that IL-21 would be required for the optimal expression of IL-12Rβ2, but not IL-12Rβ1, in CD4^+^ T cells, thereby regulating a T_H1_-biased immune profile following colon inflammation. To test this hypothesis, purified CD4^+^ T cells were either treated with recombinant murine IL-21 (rmIL-21) for 1 and 4 h or left untreated. CD4^+^ T cells treated with rmIL-21 showed a 5-fold increase in IL-12Rβ2, but not IL-12Rβ1, transcripts in a time-dependent manner (Fig. 2A). Consistent with the roles played by a functional IL-21/IL-21R signaling axis in promoting a proinflammatory phenotype in CD4^+^ T cells,^39–42^ it was shown that rmIL-21 imprinted the expression of the transcription factor T-bet transcripts in CD4^+^ T cells in a time-dependent manner (Fig. 2B). Collectively, these findings demonstrated a nonredundant function for IL-21/IL-21R signaling to selectively induce the expression of T-bet and IL-12Rβ2, but not IL-12Rβ1, in CD4^+^ T cells, thereby enhancing the responsiveness to IL-12.

### 3.4 IL-12Rβ2 downstream of IL-21/IL-21R signaling is indispensable for resistance to but dispensable for clearance of *C. rodentium* infection

**Fig. 3.**
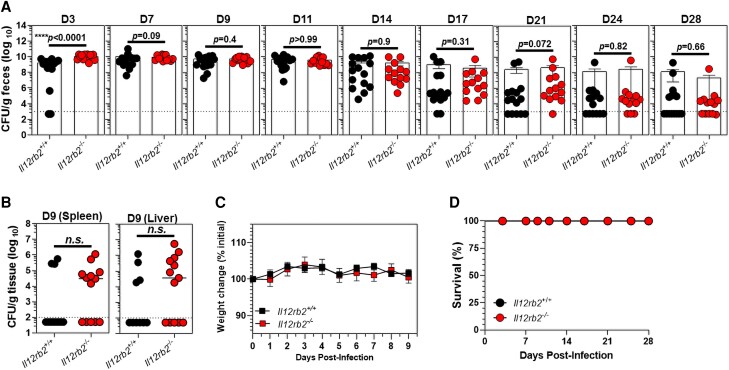
A functional IL-12Rβ2 is required for early resistance to *C. rodentium* infection but is dispensable for bacterial clearance. The (A) fecal and (B) splenic/liver bacterial burdens in *Il12rb2*^−/−^ mice and their *Il12rb2*^+/+^ littermate controls upon infection with 5 × 10^8^ CFU of *C. rodentium*, as expressed by CFU/g feces (A) or CFU/g tissue (B). Results are pooled of 2 (A) and 3 (B) independent experiments with a total of 15 (*Il12rb2*^+/+^) and 13 (*Il12rb2*^−/−^) mice/genotype (A) and 10 (*Il12rb2*^+/+^) and 13 (*Il12rb2*^−/−^) mice/genotype (B). The dashed line denotes the sensitivity of the standard culture method on MacConkey agar for feces (10^3^ CFU/g feces) and spleen/liver (10^2^ CFU/g tissue). (C) Body weight of *Il12rb2*^+/+^ mice (*n* = 8) and their *Il12rb2*^−/−^ littermate controls (*n* = 6) after *C. rodentium* infection (5 × 10^8^ CFU/mouse). Data are pooled from 2 independent experiments (D) with survival analysis of *Il12rb2*^−/−^ mice (*n* = 15) and *Il12rb2*^+/+^ littermate controls (*n* = 13) following *C. rodentium* infection. The results are shown as mean ± SEM. ****P < 0.0001 as indicated by Mann–Whitney *U* test.

The effect of the *Il12rb2* genetic ablation on the outcome of *C. rodentium*–induced colitis in *Il12rb2^−^*^/*−*^ mice and their *Il12rb2*^+/+^ littermates was further studied. *Il12rb2^−^*^/*−*^ mice were more susceptible to *C. rodentium* infection early on during the course of infection (3 days post infection [d.p.i.]) as compared with their *Il12rb2*^+/+^ littermates (Fig. 3A). However, both *Il12rb2^−^*^/*−*^ mice and their *Il12rb2*^+/+^ littermates were similarly susceptible to *C. rodentium* infection, as demonstrated by comparable fecal bacterial burdens at days 7 to 28 p.i. (Fig. 3A). Notably, both genotypes were able to efficiently eliminate *C. rodentium* infection by day 28, a time point at which immunocompetent mice normally clear the infection (Fig. 3A). *Il12rb2^−^*^/*−*^ mice and their *Il12rb2*^+/+^ littermate controls had comparable numbers of disseminated *C. rodentium* in the spleen and liver at day 9 p.i. (Fig. 3B). Weight changes in the 2 genotypes were also similar during the course of infection (Fig. 3C), and neither *Il12rb2^−^*^/*−*^ mice nor their *Il12rb2*^+/+^ littermate controls succumbed to death following *C. rodentium* infection (Fig. 3D), indicating the dispensable function of IL-12Rβ2 signaling in host immunity during colonic infection. Altogether, these findings indicated the indispensable requirement of IL-12Rβ2 signaling in the early resistance to *C. rodentium* (innate immune compartment), whereas the clearance of the infection (adaptive immune compartment) did not require functional IL-12Rβ2 signaling.

### 3.5 IL-12Rβ2 is dispensable for the expression of antigen-specific IFN-γ and IL-17A by cLP CD4^+^ T cells following *C. rodentium* infection

**Fig. 4.**
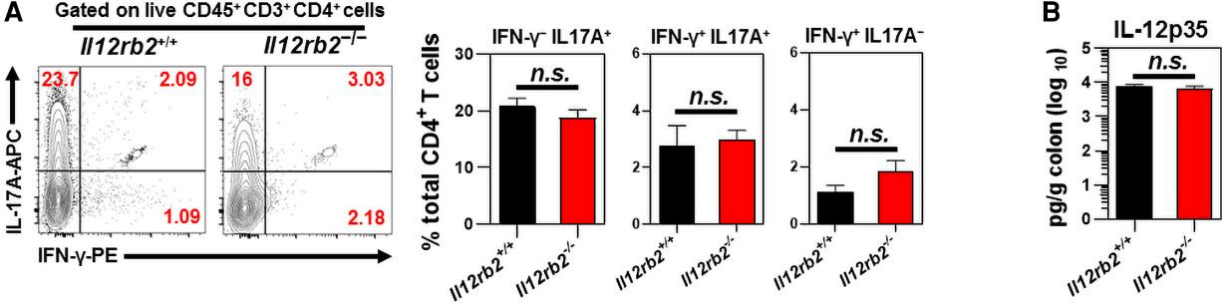
Lack of IL-12Rβ2 does not alter the ability of cLP CD4^+^ T cells to express OVA-induced IL-17A and IFN-γ following *C. rodentium* infection. (A) The intracellular expression of antigen-specific IFN-γ and IL-17A by distal cLP CD4^+^ T cells of *Il12rb2*^+/+^ mice (*n* = 5) and *Il12rb2*^−/−^ mice (n = 7) 9 d after OVA–*C. rodentium* infection. The numbers shown in the upper panels represent the percentage of CD4^+^ T cells expressing IFN-γ, IL-17A, or both. (B) Enzyme-linked immunosorbent assay was used to measure IL-12p35 concentrations in the colon culture supernatants of age- and sex-matched *Il12rb2*^+/+^ mice (*n* = 6) and *Il12rb2*^−/−^ mice (*n* = 6) 9 d after *C. rodentium* infection. Data are pooled from 2 independent experiments. The data indicate the mean ± SEM.

The *C. rodentium* colonization at the mucosal surfaces of the colon imprints a robust immune response signature, as characterized by the predominant release of IFN-γ and IL-17 by antigen-specific CD4^+^ T cells.^48–51^ This study examined whether IL-12Rβ2 deficiency impairs the intracellular expression of antigen-specific IFN-γ or IL-17 by mucosal cLP CD4^+^ T cells following *C. rodentium* infection. There were no significant differences in the expression of IFN-γ or IL-17A by distal cLP CD4^+^ T cells or those CD4^+^ T cells coexpressing both IFN-γ and IL-17A in response to chicken OVA 9 d after OVA–*C. rodentium* infection (Fig. 4A). IL-12p35 protein levels were comparable in colon culture supernatants of *Il12rb2*^+/+^ mice and their *Il12rb2^−^*^/*−*^ littermates (Fig. 4B).

These findings suggested that IL-12Rβ2 was not necessary for the expression of IFN-γ or IL-17A by antigen-specific cLP CD4^+^ T cells following *C. rodentium* infection. These observations demonstrated that the IL-12Rβ2 signaling blockade during bacterial-induced colitis did not interfere with the host's capability to eliminate infection from the colon.

### 3.6 Lack of IL-12Rβ2 does not compromise the ability of *Il12rb2^−^*^/*−*^ mice to mount the host gene expression profile in response to *C. rodentium* infection

**Fig. 5.**
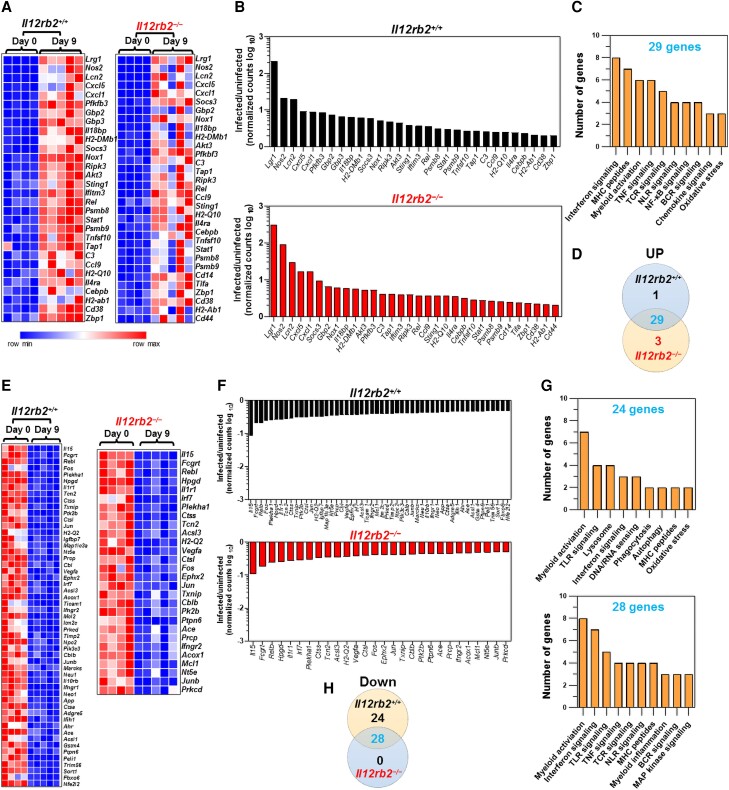
A functional IL-12Rβ2 is dispensable for the expression of immune-mediated host defense genes in the colon 9 d after *C. rodentium* infection. Heatmaps demonstrate differential genes upregulation (A, B) or downregulation (E, F) in the whole distal colons of *Il12rb2*^+/+^ mice and their *Il12rb2*^−/−^ littermates in response to infection with *C. rodentium*, as determined by Nanostring. Age- and sex-matched uninfected (*n* = 4) and infected (*n* = 5) mice/genotype were used. The analysis included genes with fold change of ≥2 (C, G) gene ontology analysis of processes enriched in whole distal colons of *Il12rb2*^+/+^ mice compared to their *Il12rb2*^−/−^ littermates in response to *C. rodentium* infection. (D, H) Venn diagrams represent the numbers of upregulated or downregulated, uniquely expressed, and overlapping genes between *Il12rb2*^+/+^ mice and their *Il12rb2*^−/−^ littermates.

A total of 30 genes (*n* = 30) involved in host immune response were upregulated (≥2-fold) in the distal colons of infected *Il12rb2*^+/+^ mice in response to *C. rodentium* infection at 9 d.p.i. (Fig. 5A to C). Notably, *Il12rb2^−^*^/*−*^ mice could also efficiently mount an approximately same set of genes (*n* = 32) after *C. rodentium* infection. Figure 5D shows that most genes (*n* = 29) overlapped between the 2 genotypes, with only 1 and 3 genes being uniquely upregulated in *Il12rb2*^+/+^ mice and *Il12rb2^−^*^/*−*^ mice, respectively. The gene ontology and pathway analysis revealed that those genes that were commonly upregulated between *Il12rb2*^+/+^ mice and *Il12rb2^−^*^/*−*^ mice following *C. rodentium* infection were mainly enriched in 10 biological pathways, with the majority of upregulated genes components of interferon signaling (*n* = 8 genes), MHC peptides (*n* = 7 genes), myeloid activation pathway, and tumor necrosis factor signaling (*n* = 6 genes each), as well as T-cell receptor signaling (*n* = 5 genes) (Fig. 5C).

Over half of the genes (28/53) that were downregulated in the colon in response to *C. rodentium* in *Il12rb2*^+/+^ mice were likewise downregulated in *Il12rb2^−^*^/*−*^ mice (Fig. 5E and F). Interestingly, 25 of 53 genes (47.1%) were uniquely downregulated in *Il12rb2*^+/+^ mice in response to *C. rodentium* 9 d after infection, but no genes were specifically downregulated in the colons of *Il12rb2^−^*^/*−*^ mice following infection (Fig. 5H). The downregulated genes in the distal colons of *Il12rb2^+^*^/*+*^ mice and their *Il12rb2^−^*^/*−*^ littermates following *C. rodentium* infection were primarily enriched in 9 and 10 biological pathways, respectively (Fig. 5G). In both genotypes, the myeloid activation pathway components were the most highly enriched pathway, followed by Toll-like receptor (TLR) signaling and interferon signaling in *Il12rb2^+^*^/*+*^ and *Il12rb2^−^*^/*−*^ mice, respectively (Fig. 5G).

### 3.7 Lack of IL-12Rβ2 signaling attenuates *C. rodentium*–induced colitis

**Fig. 6.**
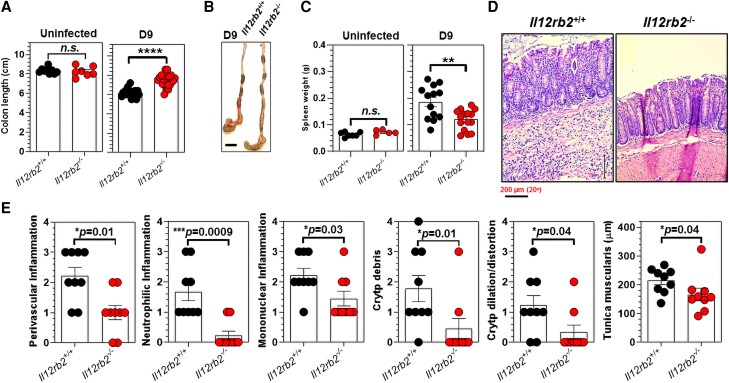
Lack of a functional IL-12Rβ2 signaling axis attenuates *C. rodentium*–induced colitis. (A) Colon lengths and (B) representative images of the cecum and colon of *Il12rb2*^+/+^ mice and *Il12rb2*^−/−^ littermates 9 d after *C. rodentium* infection. Scale bar: 1 cm. (C) Spleen masses were measured in uninfected and *C. rodentium*–infected (day 9) *Il12rb2*^+/+^ mice and their *Il12rb2*^−/−^ littermates. (D) Hematoxylin and eosin–stained histological representations of the distal colon of *Il12rb2*^+/+^ mice and their *Il12rb2*^−/−^ littermates 9 d after infection with *C*. *rodentium* (20×). Scale bar: 200 μm. (E) Histological assessments of the distal colon inflammation as well as crypt microstructural integrity and the tunica muscularis of the distal colons of *ll12rb2*^+/+^ mice (n = 9) and their *Il12rb2*^−/−^ littermates (n = 9) 9 d after infection with *C. rodentium*. Data are pooled from at least 2 independent experiments using 7 to 9 mice/genotype (A, uninfected), 16 to 20 mice/genotype (A, infected), 5 to 7 mice/genotype (C, uninfected), and 13 to 15 mice/genotype (C, infected). The data indicate the mean ± SEM. *P < 0.05, **P < 0.01, ***P < 0.001, ****P < 0.0001; Mann–Whitney *U* test.

Despite comparable bacterial burdens in the feces (Fig. 3A) and spleen/liver (Fig. 3B), *Il12rb2*^+/+^ mice had significantly shorter colon lengths (6.1 ± 0.1 cm) than their *Il12rb2^−^*^/*−*^ littermates (7.05 ± 0.152 cm) at 9 d.p.i. (Fig. 6A and B). The shorter colon lengths were not intrinsic to the lack of IL-12Rβ2 signaling, since uninfected *Il12rb2*^+/+^ mice and their *Il12rb2^−^*^/*−*^ littermates had comparable colon lengths (8.37 ± 0.132 cm vs 8.24 ± 0.196 cm, respectively) in the absence of *C. rodentium* infection (Fig. 6A). *Il12rb2*^+/+^ mice showed significantly larger spleen masses (0.193 ± 0.018 g) than *Il12rb2^−^*^/*−*^ mice (0.134 ± 0.008 g) at 9 d.p.i. Uninfected mice of both genotypes had comparable spleen sizes (Fig. 6C).

Histological examination revealed that *Il12rb2*^+/+^ mice had higher perivascular inflammation and inflammatory immune subsets in their distal colon submucosa than *Il12rb2^−^*^/*−*^ mice 9 d following *C*. *rodentium* infection (Fig. 6D). Notably, *Il12rb2*^+/+^ mice showed increased mucosal inflammation and tunica muscularis thickening, as well as increased mononuclear cell infiltration throughout the entire cLP and the formation of crypt abscesses, submucosal edema, goblet cell depletion, and crypt distortion (Fig. 6E). In contrast, *Il12rb2^−^*^/*−*^ mice displayed significantly attenuated *C. rodentium*–induced colitis, associated with a less evident accumulation of proinflammatory immune subsets and less severe microstructural alterations in the distal colon following *C. rodentium* infection (Fig. 6E).

These observations demonstrated that the lack of functional IL-12Rβ2 signaling did not affect the host's ability to efficiently control and eventually eliminate *C. rodentium* infection in the colon, yet was required for the promotion of colitis following *C. rodentium*–induced colitis in vivo. These findings also indicated the requirement of intact IL-12Rβ2 signaling to promote proinflammatory immune responses in the distal colon following *C. rodentium* infection in vivo.

## 4. Discussion

In this study, a previously less-defined function of the IL-21/IL-21R signaling in the regulation of colitis through interaction with the IL-12/IL-12Rβ2 signaling was identified. Specifically, it was found that the expression of *Ifng*, *Il12rb1*, and *Il12rb2* was impaired in colonic CD4^+^ T cells of *Il21r*^−/−^ mice during *C. rodentium*–induced colitis. However, impaired expression of *Il12rb2* in cLP CD4^+^ T cells was more noticeable than *Il12rb1*. Notably, mucosal distal cLP CD4^+^ T cells were the most predominant expressors of IL-12Rβ1 and IL-12Rβ2 in the distal colon following *C. rodentium* infection. This suggests that CD4^+^ T cells are the primary cellular targets for the immunomodulatory effects of IL-12 signaling during colitis. Furthermore, rmIL-21 increased the surface expression of IL-12Rβ2 in purified CD4^+^ T cells, whereas the surface expression of IL-12Rβ1 remained unchanged. This study also showed that the lack of functional IL-12Rβ2 signaling neither compromised the ability of the murine host to imprint a host response gene signature nor impaired the intracellular expression of antigen-specific IFN-γ and IL-17A by distal cLP CD4^+^ T cells after *C. rodentium* infection. The genetic ablation of IL-12Rβ2 led to the significant attenuation of colitis, despite its nonessential roles in eradicating *C. rodentium* infection in vivo.

Mucosal CD4^+^ T cells represent a major population in the LP of the intestinal tract, playing critical roles in the maintenance of intestinal homeostasis in both health and disease (reviewed in Shale et al.^52^ and Sheridan and Lefrançois^53^). The intestinal mucosal cLP CD4^+^ T cells predominantly consist of memory T cells with major host-protective functions during infections with a variety of enteropathogens.^54–56^ The *C. rodentium* colonization triggers a robust T_H17/_T_H1_ T helper immune response in the colon.^51,57^ This is evidenced by the infiltration and accumulation of antigen-specific CD4^+^ T cells in the cLP, which can express proinflammatory cytokines such as IFN-γ, IL-12, and IL-17.^49,51,58^ The absolute requirement of CD4^+^ T cells in protection against *C. rodentium* was further supported by the findings that mice lacking CD4^+^ T cells, but not CD8^+^ T cells, were exquisitely susceptible to infection and that the adoptive transfer of CD4^+^ T cells from infected mice upon the clearance of *C. rodentium* infection conferred protection against the same infection in naive mice.^59^

The expression of IFN-γ by antigen-specific CD4^+^ T cells protected mice from *C. rodentium* infection, whereas the conditional deletion of IL-10 in CD4^+^ T cells worsened colonic pathology as compared with their littermate controls.^60^ Despite their protective functions during *C. rodentium* infection, CD4^+^ T cells also contribute to the onset and progression of *C. rodentium*–induced colitis.^61–63^ In general, mucosal CD4^+^ T cells are considered major drivers of *C. rodentium*–induced colitis, causing goblet cell deletion after infection.^62^ To this end, various cytokines have been shown to modulate T-cell effector functions and play critical roles in the development of T-cell–mediated colitis during *C. rodentium* infection.^63^ For instance, the proinflammatory cytokine IL-6 was required for the development of a T_H17_-biased immune profile in intestinal mucosal CD4^+^ T cells following *C. rodentium* infection.^64^ However, the extent to which IL-21/IL-21R signaling contributes to the development of colitis by modulating the effector functions of CD4^+^ T cells is still unclear.

The lack of functional IL-21R signaling led to the attenuation of dextran sodium sulfate (DSS)- and *C. rodentium*–induced colitis,^13^ and the collaboration of IL-21 and IFN-γ signalings was required for the optimal expression of the interferon stimulated genes (ISGs) in CD4^+^ T cells independent of STAT3 signaling and was indispensable for protection against *C. rodentium* infection.^6^ These findings are consistent with the observations in the current study and may identify IL-21 or its cognate receptor (IL-21R) as targets for preventing colitis after bacterial-induced colitis, including infections with various strains of enteropathogenic and enterohemorrhagic *Escherichia coli* and *Clostridium difficile*. However, the direct targeting of this IL-21 or its receptor in its entirety can lead to undesirable off-target effects given the functional pleiotropy of IL-21 in controlling a wide range of immunomodulatory functions in different tissues. To this end, it would be feasible to develop strategies to harness the effector molecules downstream of IL-21/IL-21R signaling to prevent colitis without jeopardizing the host's ability against enteropathogens. The findings of the current study indicated that murine hosts lacking the IL-12/IL-12Rβ2 axis downstream of IL-21/IL-21R signaling and their littermate controls were equally susceptible to colon infection, yet *Il12rb2^−^*^/*−*^ mice exhibited attenuated *C. rodentium*–induced colitis.

IL-12 is a key cytokine required for the development of a T_H1_-biased immune profile,^22–26^ and it protects against *C. rodentium* infection.^49^ IL-12 enhances the expression of IL-12Rβ2 in CD4^+^ T cells, thereby promoting IL-12 responsiveness and facilitating the expression of T-bet and T_H1_-polarized immunity.^26,30,31^ The antibody blockade of IL-12p40/IL-12Rβ1 signaling shared with IL-23 has been employed as an effective strategy to prevent inflammation in a variety of proinflammatory conditions in humans and animal models, including CD and ulcerative colitis.^65–71^ However, the simultaneous targeting of multiple effector mediators of inflammation is most likely the key to preventing colitis (reviewed in Imam et al.^72^). To this end, the antibody blockade of IL-12p35/IL-12Rβ2 alone or in combination with existing IL-12p40/IL-12Rβ1 therapies may achieve better therapeutic outcomes in the treatment of human inflammatory conditions, including bacterial-induced colitis.

We found that the expression of *Il12rb1* was also impaired in cLP CD4^+^ T cells of *Il21r*^−/−^ mice at day 9 after *C. rodentium* infection, although to a lesser extent. It is well established that the shared p40 subunit of IL-12 and IL-23 can drive intestinal inflammation in several murine models of colitis.^66,67^ In the current study, however, the extent to which impaired *Il12rb1* expression in cLP CD4^+^ T cells contributed to attenuated colitis in *Il21r*^−/−^ mice was unclear. Further studies are required to identify the exact contribution of IL-12Rβ1 and IL-12Rβ2 in mediating the proinflammatory functions of IL-21/IL-21R signaling during colitis.

The findings of the current study provide novel insights into the mechanisms by which functional IL-21/IL-21R signaling regulates IL-12 responsiveness by promoting IL-12Rβ2 expression in CD4^+^ T cells, leading to colitis. These observations also provide novel preventive and therapeutic targets for intervening in the development of colitis and pave new avenues for future investigations.

## Data Availability

The Nanostring data are deposited in a GitHub Repository at https://github.com/shahram10/Nanostring-data.
